# Universal passivation strategy to slot-die printed SnO_2_ for hysteresis-free efficient flexible perovskite solar module

**DOI:** 10.1038/s41467-018-07099-9

**Published:** 2018-11-02

**Authors:** Tongle Bu, Jing Li, Fei Zheng, Weijian Chen, Xiaoming Wen, Zhiliang Ku, Yong Peng, Jie Zhong, Yi-Bing Cheng, Fuzhi Huang

**Affiliations:** 1 State Key Laboratory of Advanced Technology for Materials Synthesis and Processing, Wuhan University of Technology, Wuhan, 430070 P. R. China; 2 Center for Micro-Photonics, Swinburne University of Technology, Hawthorn, VIC 3122 Australia; 3 State Key Laboratory of Silicate Materials for Architectures, Wuhan University of Technology, Wuhan, 430070 P. R. China; 4 Department of Materials Science and Engineering, Monash University, Clayton, VIC 3800 Australia

## Abstract

Perovskite solar cells (PSCs) have reached an impressive efficiency over 23%. One of its promising characteristics is the low-cost solution printability, especially for flexible solar cells. However, printing large area uniform electron transport layers on rough and soft plastic substrates without hysteresis is still a great challenge. Herein, we demonstrate slot-die printed high quality tin oxide films for high efficiency flexible PSCs. The inherent hysteresis induced by the tin oxide layer is suppressed using a universal potassium interfacial passivation strategy regardless of fabricating methods. Results show that the potassium cations, not the anions, facilitate the growth of perovskite grains, passivate the interface, and contribute to the enhanced efficiency and stability. The small size flexible PSCs achieve a high efficiency of 17.18% and large size (5 × 6 cm^2^) flexible modules obtain an efficiency over 15%. This passivation strategy has shown great promise for pursuing high performance large area flexible PSCs.

## Abstract (web summary)

Uniformity and hysteresis are long lasting problems for flexible perovskite solar modules. Here Bu et al. develop a universal potassium passivation strategy to improve the quality of slot-die printed tin oxide electron transport layers and demonstrate highly efficient and hysteresis-free flexible devices.

## Introduction

Organic–inorganic metal halide perovskite solar cells (PSCs) have shown promising for commercial applications due to its low-cost and high power conversion efficiency (PCE)^1–5^. Recently, the planar structured PSCs have attracted increasing interest because of its simple structure and easy fabrication^6,7^. To improve the performance of the planar PSCs, the bottom electron transport layer (ETL) is extremely important. It should be transparent for visible light, photo-stable and compatible with the perovskites. The SnO_2_ has shown a good candidate for the efficient and stable ETL^7–9^. It can be fabricated via a low-temperature process and suitable for the flexible PSCs that possess light-weight, low-cost, weak-light photovoltaic and flexibility^10,11^. This opens possible alternative applications, such as portable power source for wearable electronics and energy source for indoor sensors. More importantly, for some special applications, such as indoor sensors, the requirement of the PCE and stability would be less demanding compared with the roof solar panels. Thus, it could accelerate commercial applications of the PSCs.

Aiming for the commercialization, fabrication of large-area PSC modules (PSCMs) has been becoming the focus of research. Although the flexible PSCs have been widely studied, the PCE of flexible PSCMs is still quite low, only 8% reported by Dagar et al. and others^12,13^. To achieve an efficient planar flexible PSC, besides the quality control of the perovskites, the charge extraction layer underneath the perovskite is more difficult to fabricate compared with that on the glass substrate. The ETL is quite thin with the thickness varying from a few nm to tens of nm. To fabricate large-area thin-film ETL without pinholes on the rough and flexible plastic substrate is of great challenge^14^. In addition, devices on plastic substrates generally limit the processing temperature below ~150 °C. Furthermore, the perovskite/ETL or HTL interfaces have been considered as serious problems which are relevant to the defects and imbalanced charge transport leading to poor performance, instability and hysteresis^15–19^. SnO_2_ ETLs can be fabricated via various methods at low temperature, including spin-coating, chemical bath deposition (CBD), atomic-layer deposition (ALD), etc. for planar PSCs^7–9,11,20–24^. It has been well accepted that the SnO_2_ could benefit the elimination of the hysteresis, a common issue in TiO_2_ ETL-based PSCs since the SnO_2_ has a deeper conduction band edge and a faster electron mobility than the TiO_2_^7,9^_._ Hysteresis-free devices have indeed been fabricated by several groups using a commercial SnO_2_ colloid precursor (Alfa Aesar, tin (IV) oxide, 15% in H_2_O colloidal dispersion, labelled as Alfa–SnO_2_)^7^. However, as the hysteresis is not only related to the charge extraction at the interface, more and more reports have shown the hysteresis still presented in the SnO_2_-based planar PSCs if without additional interfacial treatment (Supplementary Table 1)^9,10,20,25–30^. The intrinsic reason why the low-temperature processed Alfa–SnO_2_ ETL can eliminate the hysteresis is still unclear.

Here, we investigate the Alfa–SnO_2_ in detail and find that in the Alfa–SnO_2_ colloid solution there was potassium hydroxide (KOH) added to stabilize the colloids. The crucial effect of hysteresis is ascribed to the interface passivation induced by the potassium ions. Then we employ this Alfa–SnO_2_ to fabricate flexible PSCMs. By optimization of the slot-die coating of this Alfa–SnO_2_ colloid solution onto the flexible ITO/PET substrate, combining with the modulation of mixed lead halide perovskite cations, such as methylammonium (CH_3_NH_3_^+^, MA), formamidinium (CH_3_(NH_2_)_2_^+^, FA) and cesium (Cs), we obtain a 5 × 6 cm^2^ large area (aperture area of 16.07 cm^2^) flexible PSCM with high efficiency over 15% and negligible hysteresis. The Alfa–SnO_2_ has showed good performance as effective ETLs. However, considering the strong basicity (pH value is ~12) of the commercial Alfa–SnO_2_ solution, it is not suitable for production due to the alkali etching. Alternatively, we apply KOH onto the slot-die-coated the homemade SnO_2_ nanocrystals (SnO_2_ NCs) films^23^, since the potassium passivation effect could be a universal strategy to eliminate the hysteresis in the SnO_2_-based planar PSCs. A hysteresis-free high PCE of 20.50% for reverse scan (RS) and 20.46% for forward scan (FS) is obtained for rigid PSC, with dramatically improved *V*_oc_ and FF compared with the control devices which show lower PCEs of 19.27% for RS and 16.42% for FS. In addition, the small size flexible PSCs and large size flexible PSCMs also show negligible hysteresis and achieved high PCEs of 17.18% for 0.16 cm^2^ and 14.89% for 16.07 cm^2^.

## Results and Discussion

### High efficiency flexible PSCMs based on Alfa–SnO_2_ ETL

**Fig. 1:**
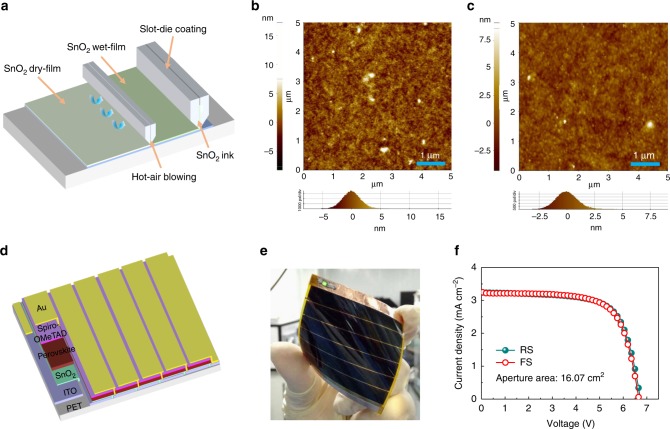
Performance of large-area flexible PSCMs based on slot-die-coated SnO_2_ substrates. **a** The schematic of slot-die coating of SnO_2_ films. **b** AFM images of the as formed SnO_2_ films without hot air assistance and **c** with hot air blowing. **d** The structure of the 6 sections series connected large-area flexible PSCMs. **e** A photograph of the flexible PSCM and **f** the corresponding *J-V* curves of the champion flexible PSCM

Slot-die coating deposition is an excellent method for large-area mass production of solution-processed films, but the solution used for the slot-die coating should be carefully adjusted, such as viscosity, volatility, wettability, toxicity, etc. Herein, the as-purchased Alfa–SnO_2_ colloidal dispersion in water was first diluted by water and then by isopropanol drop by drop, and it was then slot-die-coated onto large-area (5 × 6 cm^2^) flexible ITO/PET substrates as shown in Fig. 1a. A wind knife with hot air was attached to the slot-die head allowing the fast drying of the printed wet SnO_2_ films on ITO/PET substrates, to prevent SnO_2_ nanocrystals from aggregation in wet films, which would lead to a rough surface and pinholes in SnO_2_ films. As demonstrated in Atomic Force Microscope (AFM) images (Fig. 1b, c), the slot-die-coated SnO_2_ films quickly dried by the hot air blowing showed a smooth and compact surface with a much lower roughness (0.95 nm) compared with the naturally dried films (5.3 nm). To evaluate the homogeneity of the coating technique, an as coated 5 × 6 cm^2^ SnO_2_/ITO/PET substrate was equally cut into six pieces to measure the transmittance of SnO_2_ films (Supplementary Fig. 1a) under 550 nm light. The variation in the absorption at 550 nm was just only 3%, indicating that the film is highly uniform in such a large area. We also demonstrated the uniformity in microstructure (Supplementary Fig. 1b, c), where the SEM images show pinhole-free morphology of the SnO_2_ films deposited on the ITO/PET substrates. In addition, the transmittance of the substrates also showed an enhancement with coated SnO_2_ films due to the reduced anti-reflection.

The perovskite composition selected for this study was Cs_0.05_(FA_0.85_MA_0.15_)_0.95_Pb(I_0.85_Br_0.15_)_3_. Perovskite films were spun onto the hot-air-assisted slot-die-coated large-area pinhole-free and high-quality SnO_2_/ITO/PET substrates via a green solvent engineering method^31^, and then the whole flexible PSCMs fabrication was accomplished according to our previous report^29^. The structure of the PSCMs are illustrated in Fig. 1d, which are series connected by six subcells. And each cell is in a structure of PET/ITO/SnO_2_/perovskite/Spiro-OMeTAD/Au, respectively. Interestingly, the as-prepared flexible PSCM could light up a green LED lamp even in an indoor light, as shown in Fig. 1e, revealing its excellent photovoltaic property under weak light. The corresponding *J-V* curves of the champion PSCM are shown in Fig. 1f, with a high PCE of 15.22% for reverse scan (RS) and 15.10% for forward scan (RS), and a high *V*_oc_ of 6.727 V for RS and 6.663 V for FS, a *J*_sc_ of 3.28 mA cm^−2^ for RS and 3.28 mA cm^−2^ for FS, and a FF of 0.69 for RS and 0.69 for FS, respectively. The flexible module also exhibited outstanding stability which still remains 80% of its original efficiency after 1000 h dark storage in ambient air (~20% RH) without encapsulation (Supplementary Fig. 1d). In addition, the bending stability is also an important parameter for evaluation of the performance of the flexible devices. Herein, the slot-die-coated SnO_2_-based flexible module showed superior resistance for the mechanical bending, with no more than 30% loss even after 1800 bending cycles (Supplementary Fig. 1e). The main loss of the device performance after bending is the result of the increased FF caused by the increased *R*_s_ (Supplementary Fig. 1f) that is induced by the mechanical damage of the ITO conduction layer. This indicates that the great durability of the SnO_2_ nanoparticles composed thin films are suitable for future industrial applications of flexible PSCMs.

### Intrinsic reason for Alfa–SnO_2_ to eliminate hysteresis

**Fig. 2:**
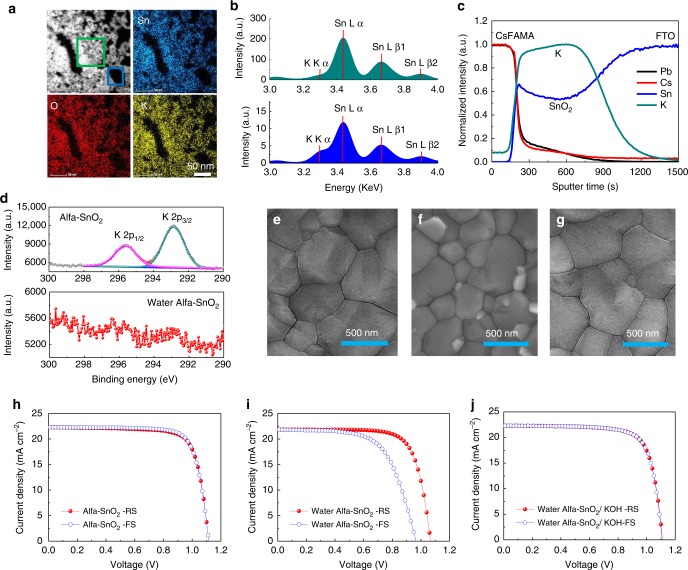
Characterization of the Alfa–SnO_2_ and corresponding performance of PSCs. **a** The STEM–EDX images of the Alfa–SnO_2_ colloidal. **b** The corresponding EDX spectra consistent to the boxed area. **c** The normalized SIMS results of the CsFAMA perovskite deposited on Alfa–SnO_2_/FTO substrate. **d** The XPS spectra of K2*p* orbital of the pristine Alfa–SnO_2_ films and Water Alfa–SnO_2_ films. **e** The SEM images of Alfa–SnO_2_ based CsFAMA perovskite film, **f** Water Alfa–SnO_2_-based CsFAMA perovskite film and **g** KOH-treated Water Alfa–SnO_2_-based CsFAMA perovskite film. **h** The *J-V* curves of Alfa–SnO_2_-based PSC, **i** Water Alfa–SnO_2_-based PSC and **j** KOH-treated Water Alfa–SnO_2_-based PSC, respectively

The hysteresis has been regarded as a serious problem in a normal planar PSC, due to its detrimental effect on the device performance and stability^15,32–34^. There are lots of reports described various types of SnO_2_ ETLs formed by different synthesis methods, deposition ways and optimization techniques, and almost all of them presented non-negligible hysteresis^9,11,26,35^. However, the PSCMs prepared from the low-temperature deposited commercial Alfa–SnO_2_ film in this work exhibited negligible hysteresis^7^. It is obviously interesting to find out the intrinsic reason for it. When carefully analysed the commercial Alfa–SnO_2_ solution, we found that the Alfa–SnO_2_ aqueous solution showed a strong basicity (pH ~12). Further investigation by the scanning transmission electron microscopy and energy dispersive X-ray spectroscopy (STEM–EDX) analysis revealed the presence of the K ions in the solution. The EDX mapping images (Fig. 2a) of the polycrystalline Alfa–SnO_2_ nanoparticles and the corresponding EDX images (Supplementary Fig. 2) of Sn, O and K elements, respectively, clearly demonstrate the existence of potassium ions. From the STEM–EDX elemental analysis, we further observed a K-rich phase located in a blank area compared with the SnO_2_ nanoparticles rich areas (Fig. 2b), which indicates that the potassium ions are mainly dissolved in the water not the SnO_2_ nanocrystals. Thus, we conclude that the potassium hydroxide (KOH) was added in the solution as a stabilizer for the SnO_2_ nanocrystals during this commercial Alfa–SnO_2_ solution preparation. Recently, potassium ions have been added into perovskite and have shown efficient passivation effect to eliminate the hysteresis^29,36^. Therefore, the hysteresis elimination effect by this Alfa–SnO_2_ should mainly be attributed to the passivation of potassium ions between the interface of SnO_2_ and perovskite.

To locate the distribution of the K ions, time of flight secondary ion mass spectrometry (TOF-SIMS) measurements were employed. Figure 2c shows the normalized SIMS data of a CsFAMA film deposited on the Alfa–SnO_2_ film, which reveals that potassium ions mainly remained in the SnO_2_ ETL and at the interface between SnO_2_ ETL and perovskite absorber layer. To further confirm the effect of the K ions, we use deionized water to bath the as-sintered Alfa–SnO_2_ films to remove the residual potassium ions (labelled as Water Alfa–SnO_2_). From the X-ray photoelectron spectroscopy (XPS) spectrum analysis, an outstanding peak of K2*p* orbital was appeared in the pristine Alfa–SnO_2_ films, while it disappeared after bathing for only10 min (Fig. 2d and Supplementary Fig. 3). This clearly indicates that the potassium ions are just blended with SnO_2_ crystals and not doped into SnO_2_ crystal lattices even after sintered at 150 °C. When the water washed SnO_2_ ETL was made into devices, as predicted it showed a severe hysteresis due to the removal of the potassium in the Alfa–SnO_2_ film, as well as a decreased performance from 19.06% for RS and 19.10% for FS to 17.40% for RS and 14.28% for FS (Fig. 2h, i). However, when the Water Alfa–SnO_2_ ETLs were re-treated with a KOH aqueous solution (10 mM, pH 12), which is labelled as the Water Alfa–SnO_2_/KOH, to introduce K ions back, the enhanced PCEs of 18.62% for RS and 18.57% for FS have been achieved with negligible hysteresis (Fig. 2j). The detailed parameters are showed in Supplementary Table 2. Actually, the perovskites deposited on the Alfa–SnO_2_ ETL and Water Alfa–SnO_2_/KOH ETL have quite similar properties, including morphology, grain size (Fig. 2e–g), XRD patterns, and UV-Vis Spectra, and corresponding EQE of devices, which are different with the perovskite deposited on the Water Alfa–SnO_2_ ETL as shown in Supplementary Fig. 4. This further confirmed the important role of the K ions at the interface on the performance of the planar PSCs, especially the hysteresis, which will be discussed in more details in the following parts.

### Potassium interface engineering for SnO_2_-based PSCs

Based on above analysis, the potassium ions at the interface would benefit the planar PSCs not only for PCEs but also the hysteresis due to the passivation effect. As discussed, the reported SnO_2_ ETLs synthesized via various methods without surface treatments almost all have the hysteresis issue. We thus treated different low-temperature processed SnO_2_ films with the KOH aqueous solution to investigate the universality of this interface passivation strategy. In addition, we also aim to replace the strong alkaline Alfa–SnO_2_ colloidal dispersion due to its disadvantages, such as strong corrosion to slot-die metal surface and poor wettability to plastic substrates (Supplementary Fig. 5).

**Fig. 3:**
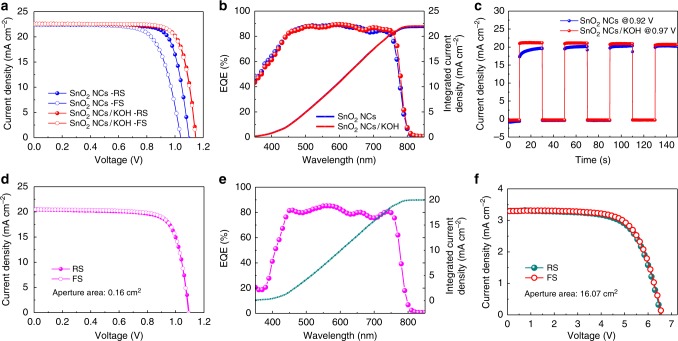
Performance of PSCs with interface potassium passivation. **a** The champion *J-V* curves of SnO_2_ NCs-based PSCs with potassium passivation or not. **b** The corresponding EQE spectra of these champion devices. **c** Steady-state output current under maximum power point with regularly turn on/off test. **d** The champion *J-V* curves of SnO_2_ NCs-based flexible PSC with potassium passivation. **e** The corresponding EQE spectra of this champion flexible device and **f** the *J-V* curves of SnO_2_ NCs-based large-area flexible PSCM

Figure 3a shows the champion *J-V* curves of the low-temperature presynthesized highly crystalline SnO_2_ nanocrystals (labelled as SnO_2_ NCs) based PSCs with or without KOH treatment. The as-synthesized SnO_2_ NCs are quite similar to Alfa–SnO_2_, with high crystallinity and negligible Cl^−^ residuals (Supplementary Figs. 2, 6). The corresponding performance of the devices treated with different concentrations of KOH solutions is shown in Supplementary Fig. 7, and the detailed *J-V* parameters are illustrated in Supplementary Table 3. The pristine device exhibits a typical *J-V* hysteresis, with a poor PCE of 16.42% under FS compared with the PCE of 19.27% under RS. When introducing an interface treatment of potassium ions by a 10 mM KOH solution treatment, an outstanding improvement of performance was obtained with a hysteresis-free high efficiency of 20.50% for RS and 20.46% for FS. The detailed parameters derived from the *J-V* curves are showed in Table 1, and a mathematical statistical distribution of these devices are also exhibited in Supplementary Fig. 8. It presents an obvious enhancement of *V*_oc_ compared with the pristine devices, which indicates the slower charge carrier recombination at the interface of PSCs. Figure 3b shows the corresponding EQE spectra of these champion devices with an integrated current density of 21.78 mA cm^−2^ for pristine devices and 21.97 mA cm^−2^ for passivated devices, respectively, which are highly consistent to the *J-V* results. In addition, the photoelectric response of passivated device based on the turn on/off test for the output current measurement under the maximum power point is showed in Fig. 3c, which is much swifter than pristine devices. Moreover, these two devices were tested under AM 1.5 G continuously for five times. It is found that the potassium passivated device showed excellent light stability compared with the non-passivated device (Supplementary Fig. 9a, b). The long-term stability of the passivated device is also much better than the non-passivated device, which decreased no more than 5% in its efficiency after 30 days when stored under dark in ambient air without encapsulation (Supplementary Fig. 9c). Besides, the widely used chemical bath deposited SnO_2_ films (labelled as CBD–SnO_2_), spin-coated SnCl_4_/isopropanol solution (labelled as Spin-SnCl_4_) with KOH treatment are also showed in Supplementary Fig. 10, and both of them exhibit negligible hysteresis and improved performance, indicating a universal passivation for different SnO_2_ substrate-based PSCs.

**Table 1:** Parameters derived from the *J-V* curves of the champion rigid PSCs based on different SnO_2_ substrates

Devices	Sweep	*V*_oc_ (V)	*J*_sc_ (mA cm^−2^)	FF	PCE (%)	Average PCE (%)	HI
SnO_2_ NCs	RS	1.099	22.45	0.78	19.27	18.55 ± 0.52	0.17
	FS	1.030	22.45	0.71	16.42	15.34 ± 0.71	
SnO_2_ NCs/KOH	RS	1.148	22.60	0.79	20.50	19.69 ± 0.41	0.01
	FS	1.146	22.60	0.79	20.46	19.48 ± 0.41	

Furthermore, this potassium passivation strategy was employed for fabrication of the flexible PSCs. Figure 3d shows the champion *J-V* curves of the interface potassium passivated flexible PSCs based on low-temperature processed SnO_2_ NCs. A comparable high efficiency device of 17.09% for RS and 17.18% for FS was obtained, with a high *V*_oc_ of 1.095 V for RS and 1.090 V for FS, a *J*_sc_ of 20.48 mA cm^−2^ for RS and 20.48 mA cm^−2^ for FS, and a FF of 0.76 for RS and 0.77 for FS, respectively. The corresponding EQE spectra is showed in Fig. 3e, which shows an integrated current density of 19.98 mA cm^−2^. Figure 3f shows the 5 × 6 cm^2^ large-area flexible PSCM based on low-temperature slot-die-coated SnO_2_ NCs, which shows a competitive performance to the commercial Alfa–SnO_2_-based flexible PSCMs. The champion device shows a high PCE of 14.47% for RS and 14.89% for FS, with high *V*_oc_ of 6.544 V for RS and 6.542 V for FS, *J*_sc_ of 3.30 mA cm^−2^ for RS and 3.30 mA cm^−2^ for FS, and FF of 0.67 for RS and 0.69 for FS, respectively.

Therefore, this interface potassium engineering is promising for hysteresis elimination and performance enhancement for low-temperature SnO_2_-based planar PSCs.

### Role of potassium ions at the interface

Potassium ions have been added into PSCs and have shown an efficient passivation effect^29,36^. Recent reports attributed the passivation mechanism to the excess halides introduced by the additive of potassium iodide^36,37^. The excess halides passivate the vacancies and thereby inhibit the halide migration and suppress the non-radiative recombination^36^. However, for the KOH treatment carried out in this work, there is not excess halides introduced as the anion is OH^−^. To confirm that the interface engineering is performed by the K^+^ ions instead of anions, we used a potassium acetate (KAc, KCH_3_COO) solution to treat the nanocrystalline SnO_2_ interface and obtained the similar effect of hysteresis elimination (Supplementary Fig. 11).

**Fig. 4:**
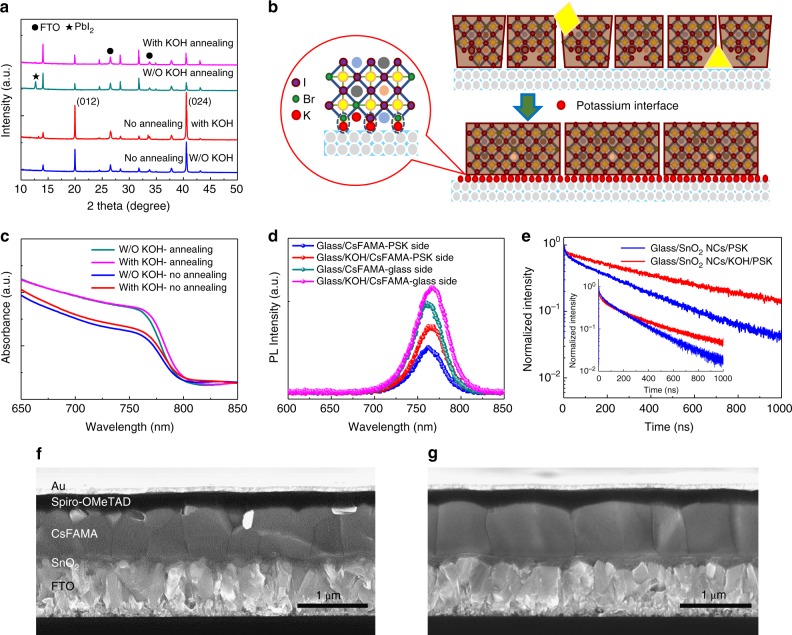
Properties of perovskite films with interface potassium passivation. **a** XRD patterns of different SnO_2_ substrates-based perovskite films with or without thermal annealing. **b** A schematic of the perovskite growth process on different substrates. **c** UV-Vis spectra of different SnO_2_ substrates-based perovskite films with/without thermal annealing. **d** The steady PL spectra of perovskites with/without interface potassium passivation. **e** The TRPL spectra of perovskites with and without interface potassium passivation. The inset plot shows the corresponding TRPL spectra when excitation laser is incident from the glass side. **f** Cross-section SEM image of the pristine perovskite device. **g** Cross-section SEM image of the perovskite device with interface potassium passivation

Thus, it is necessary to study the role of K ions at the interface. Interestingly, the K ions at the SnO_2_/perovskite interface has a great impact on the nucleation and growth of the perovskite films. When depositing the perovskite precursor onto different substrates without anti-solvent quench and annealing, we found large grains on low concentration potassium-treated substrate (Supplementary Fig. 12), but when a further increase of KOH on the surface of the substrate, too many nuclei formed, consuming too much solute to allow the nuclei to grow, and leading to small size grains in the final microstructure of the film. Therefore, an annealing-free porous film consisting of small size perovskite grains was found on the untreated SnO_2_ ETL (PSK-NM) even after anti-solvent quench, but an annealing-free dense film with large grains formed on the KOH-treated SnO_2_ ETL (PSK-KOH) film (Supplementary Fig. 13). Furthermore, after thermal annealing at 120 °C, the former showed a lot of PbI_2_ crystals on the perovskite film surface, but the latter appeared a much clear and smooth surface with larger grains (Supplementary Fig. 14). The corresponding XRD result confirmed the residual PbI_2_ in the annealed PSK-NM perovskite film (Fig. 4a). It’s also noticed that before annealing the perovskite crystals in both samples showed a clear orientation of the (012) plane parallel to the substrate, but the preferential orientation disappeared after annealing (Fig. 4a).

Considering the strong ionic bond of KBr, it is possible that the K ions introduced from KOH could readily form KBr by substitutional reaction with the Br ions at the surface of the perovskite film^38^. The KBr^−^ rich interface would passivate the halide vacancies at the interface to promote the high performance including the hysteresis elimination. Furthermore, the strong dipole of KBr would preferentially link PbX_2_ and act as nucleus for perovskite formation, as proposed in Fig. 4b. The leaching of the Br^−^ from the perovskite film then resulted in more I^−^ rich perovskites to form, which is consistent with the red-shift of the UV-Vis spectra (Fig. 4c), also confirmed by the EQE in the completed devices (Supplementary Fig. 7). In addition, the XRD also shows a slight shift to the lower angle (Supplementary Fig. 15), indicating the expansion of the lattice by incorporation of more I^−^ in the crystal. This can also explain why the perovskite deposited on the KOH-treated ETL has much less free PbI_2_ after annealing. The K^+^ nucleus facilitate the perovskite crystal growth even without thermal annealing. Such annealing-free PSCs exhibit excellent photovoltaic performance of high PCEs of 18.16% for RS and 18.15% for FS, much higher than the non-treated device (11.74% for RS and 7.63% for FS) as shown in Supplementary Fig. 16 and Supplementary Table 4.

After the thermal annealing, the PSK-KOH perovskite shows a better quality, as revealed by the steady-state PL spectra (Fig. 4d). Under the same excitation and detection conditions, the PSK-KOH perovskite has a stronger PL intensity than the PSK-NM perovskite, demonstrating the high quality of the whole perovskite film. This is further supported by time-resolved PL measurement using time correlated single photon counting (TCSPC) (Fig. 4e). When the laser is incident from the perovskite side, the bottom ETL has a very little effect on the photogenerated carriers and thus the PL decay is dominated by the carrier recombination dynamics in the perovskite. The KOH-treated sample shows much longer lifetime, confirming relatively low non-radiative recombination of the perovskite. The cross-section SEM images (Fig. 4f, g) show that in the normal direction of the film there is single-grain, which would promote the fast charge transport. When the excitation laser is incident from the glass side (the inset plots in Fig. 4e), the KOH-treated ETL enhanced the interface charge extraction. Generally, in this case the PL decay is controlled by three components, bulk perovskite recombination, interface recombination through surface trapping and carrier extraction by ETL. It is reasonable to assume the interface recombination is not significant because of the corresponding high efficiency solar cells. To quantitatively describe this process, we have fit the PL decay curves by the two-exponential function. Two PL decay curves are well fitted and the fitting parameters are listed in Supplementary Table 5. The short time constant (*τ*_1_) can be ascribed to electron extraction. The shorter constant 12.02 ns is acquitted for the treated ETL, relative to the longer one in untreated ETL. It should note the weight of the faster component significantly increases from 35 to 45%, clearly indicating the enhanced electron extraction. The slow component (*τ*_2_) is ascribed to bulk recombination, significantly increased from 226.83 ns for untreated sample to 273.68 ns for treated sample, indicating the improved quality in perovskite by the KOH treatment. The perovskite also showed a lower surface potential difference with potassium passivation (Supplementary Fig. 17), therefore, the high quality of the perovskite along with the passivated traps at the interface would benefit the performance of the devices including the hysteresis^39^.

### Reduced defects at the interface for the reduced hysteresis

**Fig. 5:**
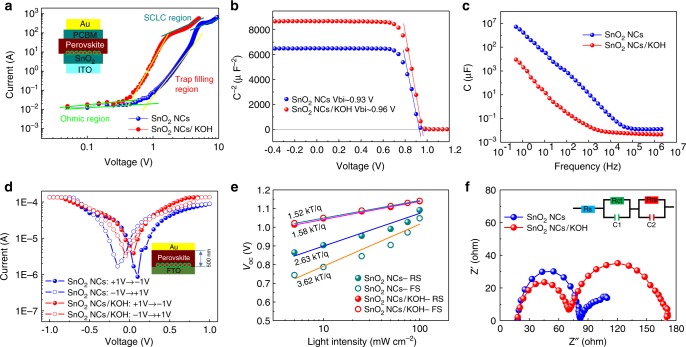
Mechanism analysis of interface potassium passivation. **a** Dark *J–V* characteristics of electron-only devices with and without interface potassium passivation. The inset shows the structure of the corresponding electron-only device. **b** Mott–Schottky plots of PSCs with and without interface potassium passivation. **c** Capacitance–frequency plots of PSCs with and without interface potassium passivation. **d** The logarithmic *I-V* plots of the FTO/CsFAMA/Au and FTO/KOH/CsFAMA/Au device, respectively. The inset shows the structure of the corresponding device. **e** The *V*_oc_ *vs*. Light Intensity curves of PSCs with and without interface potassium passivation and **f** the typical Nyquist plots of PSCs with and without interface potassium passivation. The inset shows the equivalent circuit diagram

In order to further explore the interface effects of potassium on the devices, the electron-only devices of a glass/FTO/SnO_2_/with or without KOH/perovskite/PCBM/Au architecture were fabricated for accurately assess the trap density in these different devices. The dark *J–V* characteristics were measured to obtain the electron densities for all these devices. Figure 5a shows the dark *J–V* curves of these electron-only devices with/without interface potassium treatment. The space-charge-limited-current (SCLC) technique was used to estimate the trap densities of these devices^40,41^. The linear region at low bias voltage reveals an ohmic-type response, and a marked increase of the current injection follow the increase of the bias voltage at the intermediate region is identified as the trap-filling process. The kink point between these two regions is defined as the trap-filling limit voltage (*V*_TFL_), and therefore the trap density (*N*_d_) in devices can be calculated from the following relation^41^:

$$V_{{\mathrm{TFL}}} = eN_{\mathrm{d}}L^2/2\varepsilon \varepsilon _0,$$

where *e* presents the elementary charge, *ε* stands for the relative dielectric constant, *ε*_0_ is the vacuum permittivity and *L* is the thickness of the perovskite films. The electron trap density in the potassium passivated device is calculated to be 4.42 × 10^15^ cm^−3^, which is much lower than that for the pristine device (8.86 × 10^15^ cm^−3^). This reduced trap density can be attributed to the high quality of the perovskite crystals induced from the potassium passivation, which also contributes to reduced hysteresis in the PSCs.

Figure 5b illustrates the Mott–Schottky (M-S) plots of these PSCs at 10 kHz. The interface engineered PSC demonstrates an increased build-in potential (*V*_bi_) of 0.96 V and a corresponding decreased carrier density (*N*_d_) of 1.01 × 10^16^ cm^−3^, compared with the non-treated solar cell (0.93 V and 1.18 × 10^16^ cm^−3^, respectively), which indicates fast charge collection and less carrier accumulation at the interface^42–44^. In addition, a capacitance–frequency (*C*-*f*) plots is also conducted as shown in Fig. 5c. Generally, the capacitance of the devices in the low-frequency regime may be ascribed to ionic migration to the contact interface^44–46^. The perovskite device with interface potassium treatment presents decreased capacitance compared with the pristine device, which in turn decreases the hysteresis of the device. Besides, we characterized the *J*-*V* properties by scanning from negative bias to positive bias, and then re-scan from positive bias to negative bias of these two perovskite films with/without potassium passivation. The device architecture is showed in Fig. 5d, and the corresponding logarithmic plots indicate the misalignment of the perovskite films under contrast sweep direction. A significant misalignment of the non-passivated perovskite films shows more moveable ions compared with the potassium passivate perovskite films.

The change of the *V*_oc_ of the PSCs made from the SnO_2_ ETL with/without the KOH treatment was also measured under different light intensity (Fig. 5e). It is found that a more monotonic increase in *V*_oc_ with the light intensities under either RS or FS of the potassium passivated device compared with the non-passivated devices. As is well known, the deviation of the slope from unity *kT*/*q* (where *k* is the Boltzmann constant, *T* represents the absolute temperature and *q* denotes the elementary charge) suggests a trap-assisted recombination in solar cells^32,47^. The PSC with potassium interface passivation shows a slope of 1.52 *kT*/*q*, which is much lower than the non-passivated device (2.63 *kT*/*q*) (Fig. 5e). It indicates a substantially reduced trap-assisted recombination and efficient charge extraction resulting from suppressed recombination. This is also evident from the electrochemical impedance spectroscopy (EIS) measurement, a useful technique that reveals the potential carrier transport behaviours in the PSCs^48–50^. The Nyquist plots of these devices with interface passivation or non-passivation were obtained under AM 1.5 G sunlight illumination and with an applied bias voltage of 1.0 V close to the *V*_oc_ as shown in Fig. 5f. There are two different semicircles which are located at different frequency ranges in the Nyquist plots. Generally, the high-frequency component is the signature of the charge transport resistance (*R*_ct_), and the low-frequency one corresponds to the charge recombination resistance (*R*_rec_) occurring at the interfaces^51,52^. The fitted *R*_ct_ and *R*_rec_ of these devices were plotted with the applied forward biases (Supplementary Fig. 18). The *R*_ct_ value in the interface potassium passivated perovskite is decreased, while the *R*_rec_ is much higher compared with the pristine one. Then the EIS data obviously shows the improved charge transport, as well as the reduced recombination in PSCs with interface potassium passivation.

### Conclusion

In summary, we reviewed the recently reported works about the PSCs using various SnO_2_ as the electron transporting materials and conclude that the hysteresis phenomenon is commonly observed in all these devices, except for additional treatments. Based on our analysis, we provided a facile interface passivation strategy with potassium treatments for SnO_2_ ETLs to obtain high efficiency, hysteresis-free, stable and low temperature fabricated planar PSCs on glass and flexible PSCs as well as flexible PSCMs on plastics. We achieved an outstanding performance of rigid planar PSC with PCE of 20.50% for RS and 20.46% for FS and a flexible PSC with PCE of 17.09% for RS and 17.18% for FS on a small aperture area of 0.16 cm^2^ after passivation, respectively. In addition, we also obtained a high PCE of 15.22% for a flexible PSCM based on slot-die-coated Alfa–SnO_2_ substrate and a PCE of 14.89% for homemade SnO_2_ NCs. The additional of the K ions in the interface forms KBr that thus passivates the surface defects of the perovskite to promote the high performance and hysteresis-free properties. The positive effects brought by the potassium ions are promising for pursuing high performance and stable large area PSCMs. This universal SnO_2_ surface engineering strategy opens an effective way to fabricate the high quality SnO_2_ ETL for the planar PSCs and PSCMs.

## Methods

### Materials

The SnO_2_ colloidal dispersion (tin (IV) oxide, 15% in H_2_O colloidal dispersion) was purchased from Alfa Aesar. The SnCl_2_·2H_2_O, SnCl_4_·5H_2_O, anhydrous SnCl_4_ solution and KOH were purchased from Aladdin. Lead iodide (PbI_2_) and lead bromine (PbBr_2_) were purchased from TCI. Formamidinium iodide (FAI) and methylammonium bromine (MABr) were purchased from Lumtec Technology Corp., Taiwan. The Spiro-OMeTAD was purchased from Xi’an Polymer Light Technology Corp. All other chemicals were purchased from Sigma-Aldrich or Alfa Aesar and used as received unless specified.

### Preparation of the rigid and flexible substrates

The rigid FTO/glass or flexible ITO/PET substrates were first etched using a femtosecond laser machine. Then they were cleaned through ultrasonic cleaning by detergent, pure water, and ethyl alcohol for 20 min, respectively. After that, the dry-air gas flow was employed to dry the films and then treat the films by plasma for 5 min before use.

### Preparation of the SnO_2_ films

Alfa–SnO_2_ films: The as purchased SnO_2_ colloidal dispersion was diluted by water (1:3 wt), and then spin-coated onto the clean FTO/glass substrates at 3000 rpm for 30 s and repeated for two times, then annealed at 150 °C for 1 h. For flexible devices, the SnO_2_ NCs/isopropanol solution was spin-coated onto the clean ITO/PET substrates, followed by the annealing at 140 °C for 1 h.

Water Alfa–SnO_2_ films: The diluted Alfa–SnO_2_ solution was spin-coated on the bare glasses and sintered at 150 °C for 30 min. After cooling down, a proper amount of deionized water was added into a vessel to bath the as-sintered Alfa–SnO_2_/glasses for different time.

SnO_2_ NCs films: The SnO_2_ NCs were achieved by a low temperature hydrothermal method. The anhydrous SnCl_4_ solution was diluted to 0.15 M by iced deionized water, and then kept in an oven at 90 °C. After hydrothermal treatment for an hour, the solution was washed by diethyl ether/isopropanol mixed solution for three times and followed by centrifugal separation. The centrifuged solid was re-dissolved in isopropanol with a concentration of 10 mg mL^−1^. Lastly, the SnO_2_ NCs/isopropanol solution was spin-coated onto the clean FTO/glass substrates at 3000 rpm for 30 s and repeated for three times, then annealed at 180 °C for 1 h. For flexible devices, the SnO_2_ NCs/isopropanol solution was spin-coated onto the clean ITO/PET substrates, followed by annealing at 140 °C for 1 h.

Spin-coated SnO_2_ films: The spin-coated SnO_2_ films were fabricated using SnCl_4_•5H_2_O/isopropanol precursor. A 50 µL of 0.075 M SnCl_4_•5H_2_O/isopropanol precursor was spun onto the clean FTO/glass substrates at a spin rate of 3000 rpm for 30 s. The films were then annealed at 180 °C for 1 h. After cooling down, the as-sintered SnO_2_ substrates were soaked into a hot water bathing at 90 °C for 1 h. Finally, the films were annealed at 180 °C for 30 min.

CBD deposited SnO_2_ films: The CBD–SnO_2_ films were achieved by chemical bath deposition method. 5 g urea was firstly dissolved into 400 mL deionized water, followed by the addition of 100 µL thioglycollic acid and 5 mL HCl (37 wt%). Finally, the SnCl_2_·2H_2_O powder was dissolved in the solution at a concentration of 0.012 M and then stored in fridge for 3 days before use. The as-cleaned FTO glass was soaked into the diluted SnCl_2_·2H_2_O solution (0.002 M) for 2 h at 70 °C and then washed by deionized water and dried by gas gun blowing. The CBD process was repeated for three times in order to achieve the desired thickness, and followed by annealing at 180 °C for 1 h.

KOH-treated SnO_2_ films: The KOH powder was dissolved in water at a concentration of 5 mM, 10 mM, 20 mM, 50 mM and 100 mM respectively, and then spin-coated onto the SnO_2_ films at 3000 rpm for 30 s, followed by annealing at 100 °C for 10 min.

Slot-die-coated SnO_2_ onto large area flexible plastic substrates: The SnO_2_ films were deposited under ambient conditions using a homemade slot-die setup on a 3D mobile platform. The as-purchased Alfa–SnO_2_ colloidal dispersion in water was diluted by H_2_O firstly, and then diluted using isopropanol (IPA), drop by drop, under continuous stirring, with the final volume ratio of H_2_O/IPA to 1:1. The diluted concentration was about 10 mg mL^−1^, and the synthesized SnO_2_ NCs were also diluted by isopropanol with a concentration about 10 mg mL^−1^. The diluted SnO_2_ solution was deposited on a clean ITO-PET substrate by slot-die coating for three cycles, followed by annealing at 140 °C for 1 h. The distance between substrate and slot-die lip was 0.25 mm (*Z* = 0.25 mm). The coating speed was controlled by a built-in controller of the 3D mobile platform while the solution flow through the slot-die head was controlled by a syringe pump (stage speed *V* = 15 mm s^−1^, the solution flow rate = 58 L s^−1^).

### Preparation of the mixed perovskite precursor

The CsFAMA mixed perovskite precursor was prepared by dissolving 1.3 M organic cation (0.85 FAI and 0.15 MABr) and 1.4 M mixture of metal lead salts (0.85 PbI_2_ and 0.15 PbBr_2_) in a mixture solvent of DMF/DMSO (4:1, by volume), and then a 34 µL CsI solution (pre-dissolved as a 2 M stock solution in DMSO) was added to achieve the desired Cs_0.05_(FA_0.85_MA_0.15_)_0.95_Pb(I_0.85_Br_0.15_)_3_ perovskite precursor solution with proper excess lead halide.

### Preparation of the Spiro-OMeTAD solution

The Spiro-OMeTAD solution was prepared by firstly dissolving 73 mg Spiro-OMeTAD in 1 mL chlorobenzene. After that, 18 µL Li-TFSI (from 520 mg mL^−1^ stock acetonitrile solution) and 29 µL FK209 (300 mg mL^−1^ stock acetonitrile solution) and 30 µL 4-tert-butylpyridine, were added into the solution. The solution was continuously stirring for 10 min before use.

### Device fabrication

The mixed perovskite precursor was spin-coated on these different SnO_2_ substrates at a spin rate of 6000 rpm for 30 s with accelerated speed of 1000 rpm. At the last 5th second, a 100 µL green anti-solvent of ethyl acetate was drop-coated, and then the as deposited films were annealed at 120 °C for 45 min. After cooling down, the Spiro-OMeTAD solution was spin-coated on the perovskite films at 3000 rpm for 30 s. Finally, 80-nm thick of gold was deposited using thermal evaporation to complete the whole devices. The deposition of large area perovskite films was referred to a previous report^29^. First, a femtosecond laser was used to scribe the 5 × 6 cm^2^ cleaned ITO/PET substrate to form the module substrate with six strips. Then different SnO_2_ precursors were deposited on the cleaned module substrate via a slot-die coating method. The perovskite and Spiro-OMeTAD films were fabricated by spin-coating via the same procedure as the small devices except with more solvent dosage. Lastly, the device was laser-scratched again to form the series-connected module before deposition of the gold.

### Characterizations

The SnO_2_ nanoparticles were characterized using a high resolution TEM (Talos F200S, Thermo Fisher, USA). The surface morphologies and microstructures of SnO_2_ films, perovskite films and the cross-sectional structure of these PSCs were investigated using a field-emission scanning electron microscopy (FESEM, Zeiss Ultra Plus). The SnO_2_ and perovskite films were also characterized by TOF-SIMS (TOF.SIMS 5-100, ION-TOF GmbH), UV-Vis spectrometer (lambda 750 S, PerkinElmer), X-ray diffractometer (XRD, D8 Advance), atomic force microscope (AFM) and kelvin probe force microscope (KPFM) (SPM9700, Shimadzu, Japan), respectively. The thickness of the perovskite, SnO_2_ and FTO tested in TOF-SIMS are approximately 500 nm, 100 nm and 800 nm, respectively. The EIS measurements of these PSCs were carried out by an EC-lab (SP300). The *J*-*V* curves of these PSCs were measured using a Keithley 2400 source meter at room environment. The light source was a solar simulator (Oriel 94023 A, 300 W) to match AM 1.5 G. The intensity of the light was 100 mW cm^−2^ calibrated by a standard silicon reference solar cell (Oriel, VLSI standards). All the devices were tested using a black metal aperture with a defined active area of 0.16 cm^2^ for the small devices and 16.07 cm^2^ for the large 5 cm × 6 cm PSCMs, respectively.

## Electronic supplementary material

Supplementary Information

Solar Cells Reporting Summary

## Data Availability

The data that support the findings of this study are available from the corresponding authors on reasonable request.
