# Supplementary material for: Universal passivation strategy to slot-die printed SnO2 for hysteresis-free efficient flexible perovskite solar module
Source: Nat Commun. 2018 Nov 2;9:4609. doi: 10.1038/s41467-018-07099-9 (PMC6214926; doi:10.1038/s41467-018-07099-9)
Supplement: Supplementary file 1 — Supplementary Information [file 41467_2018_7099_MOESM1_ESM.pdf]

nature research

Corresponding author(s): Tongle Bu, Jing Li, Fei Zheng, Weijian Chen, Xiaoming Wen, Zhiliang Ku, Yong Peng, Jie Zhong, Yi-Bing Cheng and Fuzhi Huang

Solar Cells Reporting Summary

Nature Research wishes to improve the reproducibility of the work that we publish. This form is intended for publication with all accepted papers reporting the characterization of photovoltaic devices and provides structure for consistency and transparency in reporting. Some list items might not apply to an individual manuscript, but all fields must be completed for clarity.

For further information on Nature Research policies, including our data availability policy, see Authors & Referees.

Experimental design

Please check: are the following details reported in the manuscript?

1. Dimensions

Item	Yes	No	Details
Area of the tested solar cells	☒	☐	It can be found in the part of 'Methods'.
Method used to determine the device area	☒	☐	The method can be found in the part of 'Methods'.

2. Current-voltage characterization

Item	Yes	No	Details
Current density-voltage (J-V) plots in both forward and backward direction	☒	☐	It can be found in both text and figures: Fig.1-3.
Voltage scan conditions<br>*For instance: scan direction, speed, dwell times*	☒	☐	It can be found in the part of 'Methods'.
Test environment<br>*For instance: characterization temperature, in air or in glove box*	☒	☐	It can be found in the part of 'Methods'.
Protocol for preconditioning of the device before its characterization	☐	☒	The device is stable and does not need special precondition.
Stability of the J-V characteristic<br>*Verified with time evolution of the maximum power point or with the photocurrent at maximum power point; see ref. 7 for details.*	☒	☐	It can be seen in Fig. 3c.

3. Hysteresis or any other unusual behaviour

Item	Yes	No	Details
Description of the unusual behaviour observed during the characterization	☒	☐	Fig. 3a described the behavior of hysteresis.
Related experimental data	☒	☐	Fig. 3a, Table 1

4. Efficiency

Item	Yes	No	Details
External quantum efficiency (EQE) or incident photons to current efficiency (IPCE)	☒	☐	Fig. 3b, Fig. 3d
A comparison between the integrated response under the standard reference spectrum and the response measure under the simulator	☒	☐	Fig. 3d, Fig. 3e
For tandem solar cells, the bias illumination and bias voltage used for each subcell	☐	☒	No tandem solar cells are involved here.

5. Calibration

Item	Yes	No	Details
Light source and reference cell or sensor used for the characterization	☒	☐	It can be found in the part of 'Methods'.
Confirmation that the reference cell was calibrated and certified	☒	☐	It can be found in the part of 'Methods'.

November 2017

Calculation of spectral mismatch between the reference cell and the devices under test

- [ ] Yes
- [x] No

The solar simulator is 3A grade, i.e., the spectral mismatch is below 2%.

6. Mask/aperture

Size of the mask/aperture used during testing

- [x] Yes
- [ ] No

It can be found in the part of 'Methods'.

Variation of the measured short-circuit current density with the mask/aperture area

- [x] Yes
- [ ] No

Table 1

7. Performance certification

Identity of the independent certification laboratory that confirmed the photovoltaic performance

- [ ] Yes
- [x] No

We are not claiming the record efficiency.

A copy of any certificate(s)
Provide in Supplementary Information

- [ ] Yes
- [x] No

We are not claiming the record efficiency.

8. Statistics

Number of solar cells tested

- [x] Yes
- [ ] No

Supplementarty Figure 8

Statistical analysis of the device performance

- [x] Yes
- [ ] No

Supplementarty Figure 8

9. Long-term stability analysis

Type of analysis, bias conditions and environmental conditions
For instance: illumination type, temperature, atmosphere humidity, encapsulation method, preconditioning temperature

- [x] Yes
- [ ] No

Supplementary figure 9
