# Supplementary material for: Universal passivation strategy to slot-die printed SnO2 for hysteresis-free efficient flexible perovskite solar module
Source: Nat Commun. 2018 Nov 2;9:4609. doi: 10.1038/s41467-018-07099-9 (PMC6214926; doi:10.1038/s41467-018-07099-9)
Supplement: Supplementary file 2 — Solar Cells Reporting Summary [file 41467_2018_7099_MOESM2_ESM.pdf]

Universal Passivation Strategy to Slot-die Printed SnO_2 for Hysteresis-free Efficient Flexible Perovskite Solar Module

Bu et al.

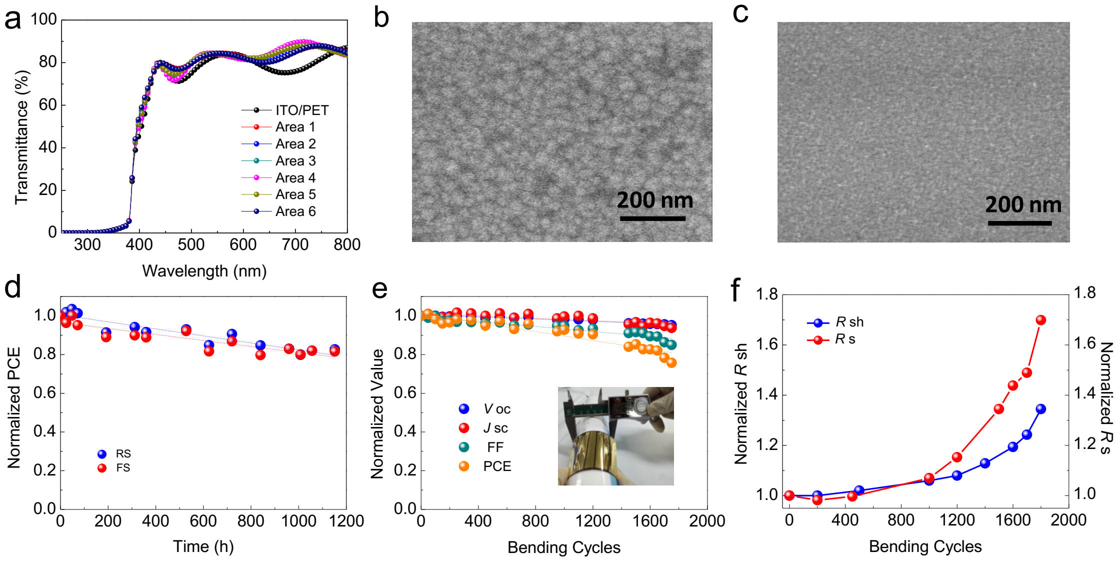

Supplementary Figure 1 a Transmittance spectra of slot-die printed SnO_2 films. SEM images of **b** bare ITO/PET substrate and **c**SnO_2 films on ITO/PET substrate. **d** Normalized PCE *vs.* aging time plots of flexible PSCMs. **e** Bending stability of flexible PSCMs and **f**the plots of calculated R_{sh} and R_s of flexible PSCMs *vs*. bending cycles

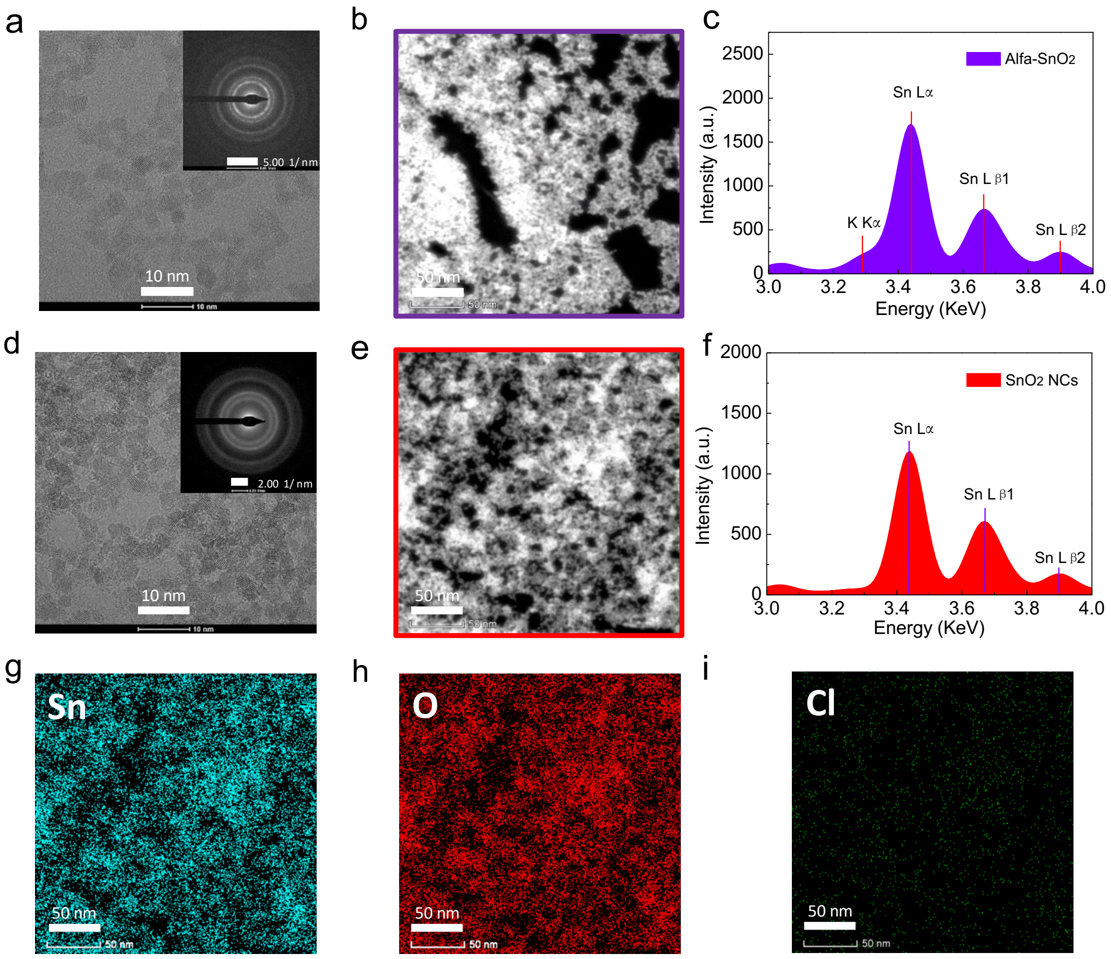

Supplementary Figure 2 a and **b** TEM images of colloidal Alfa-SnO_2. **c** The corresponding EDX spectra of Alfa-SnO_2. **d** and **e** TEM images of synthesized SnO_2 NCs. **f** The corresponding EDX spectra of synthesized SnO_2 NCs. The STEM–EDX images of the synthesized SnO_2 NCs: **g** Sn, **h** O, and **i** Cl

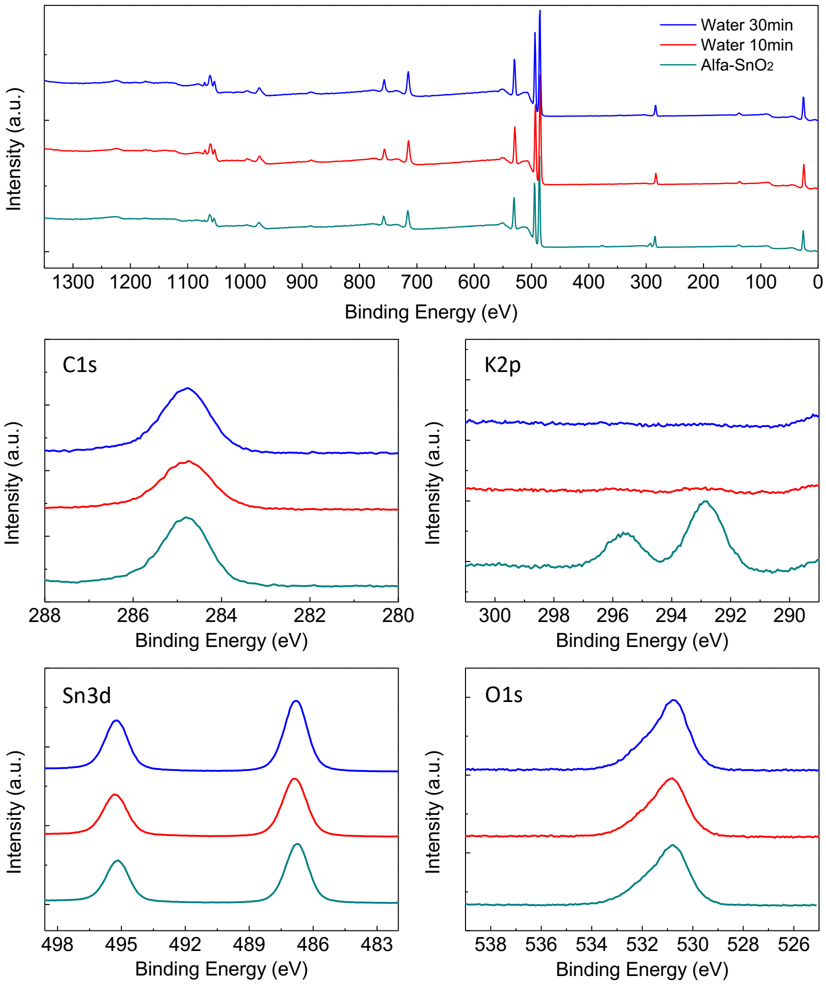

Supplementary Figure 3 XPS spectra of the Alfa-SnO_2 films with or without water washing

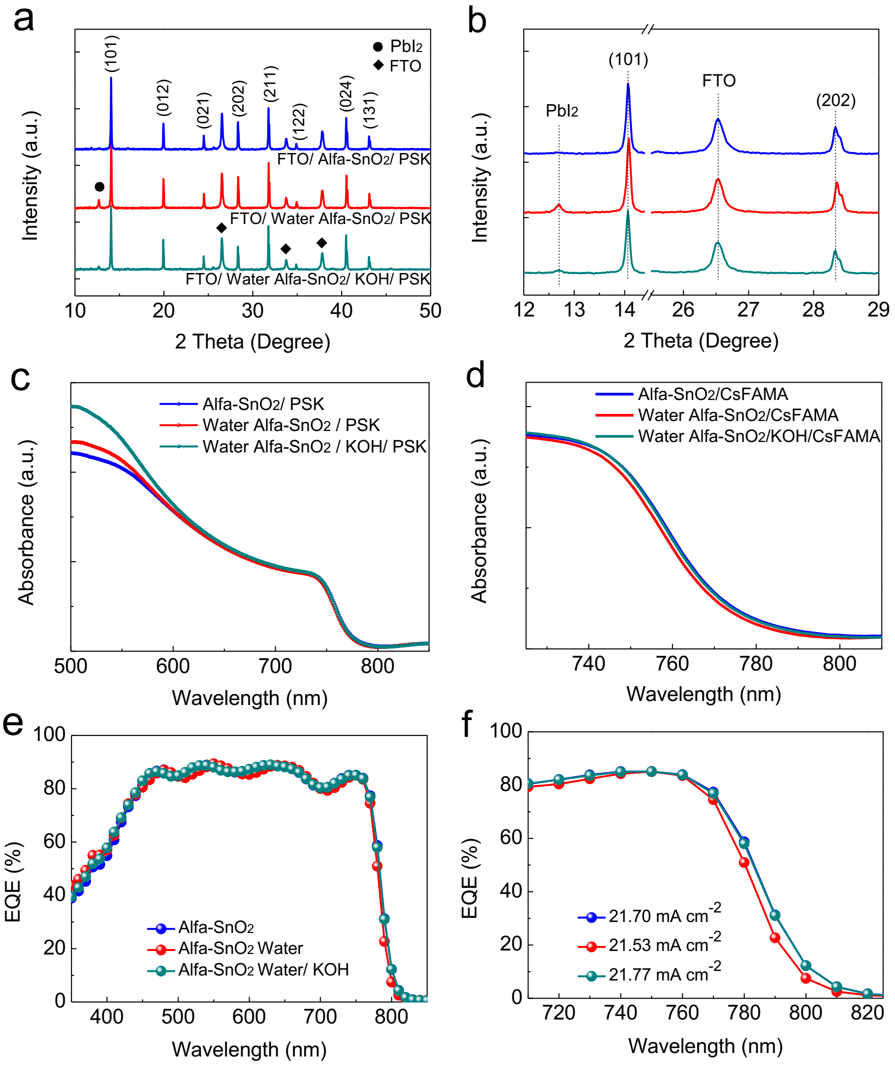

Supplementary Figure 4 **a** and **b** XRD pattern of CsFAMA perovskites deposited on the Alfa-SnO_2, Water Alfa-SnO_2, and Water Alfa-SnO_2/KOH films. **c** and **d** UV-Vis spectra of CsFAMA perovskites deposited on the Alfa-SnO_2, Water Alfa-SnO_2, and Water Alfa-SnO_2/KOH films. **e** and **f** EQE spectra of different SnO_2 based perovskite devices

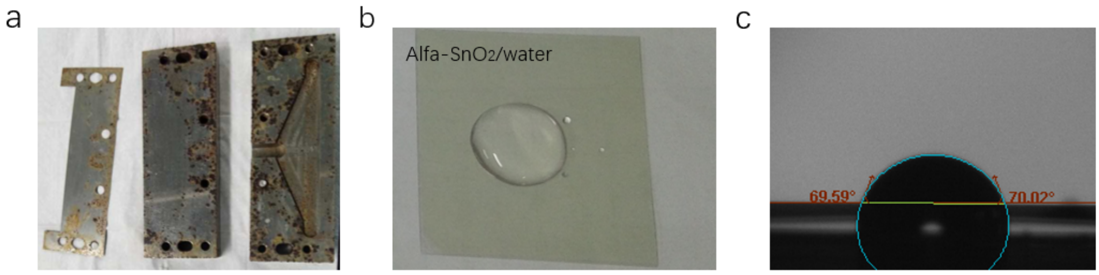

Supplementary Figure 5 **a** Pictures of metal slot-die components corroded by the Alfa-SnO_2. **b** The Alfa-SnO_2 solution diluted in water on a plasma treated ITO-PET substrate and **c** the corresponding contact angle

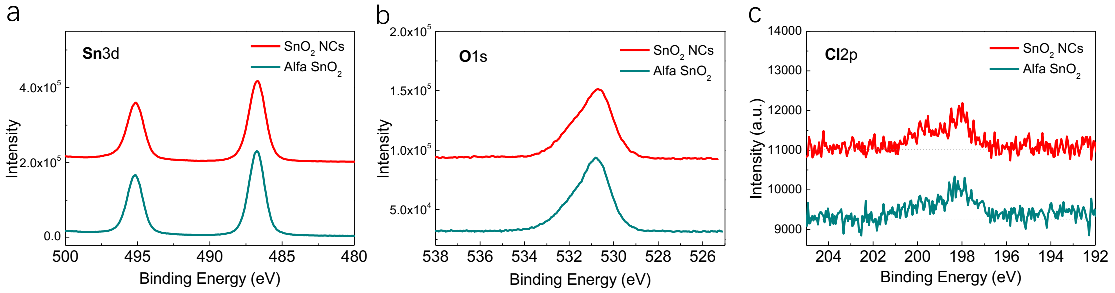

Supplementary Figure 6 XPS spectra of the Alfa-SnO_2 and SnO_2 NCs films

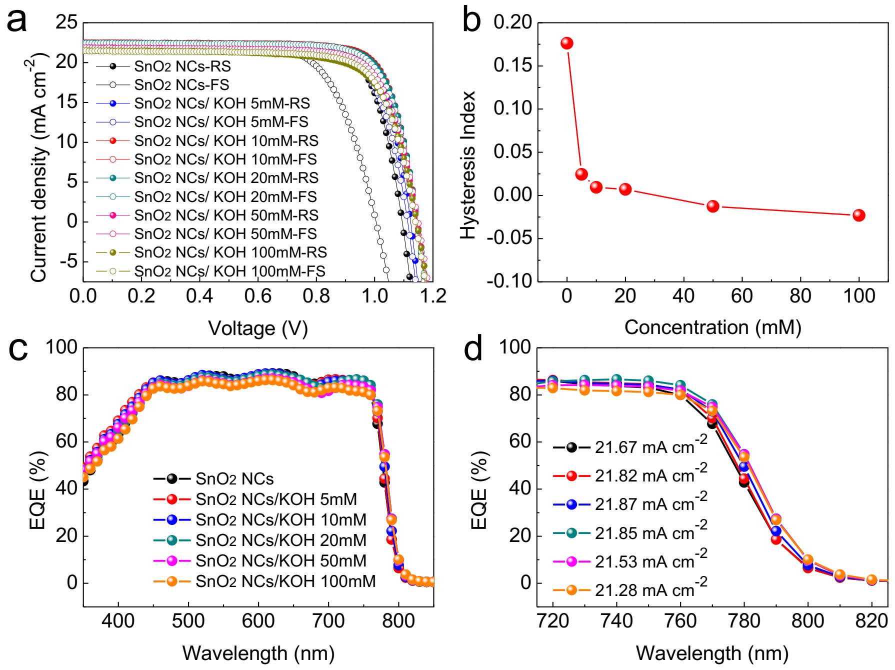

Supplementary Figure 7 **a** Typical *J-V* curves of PSCs based on different concentration of KOH solution treated SnO_2 NCs. **b** The corresponding HI calculated from the typical *J-V* curves of PSCs. **c** and **d** The corresponding EQE spectra of these different PSCs

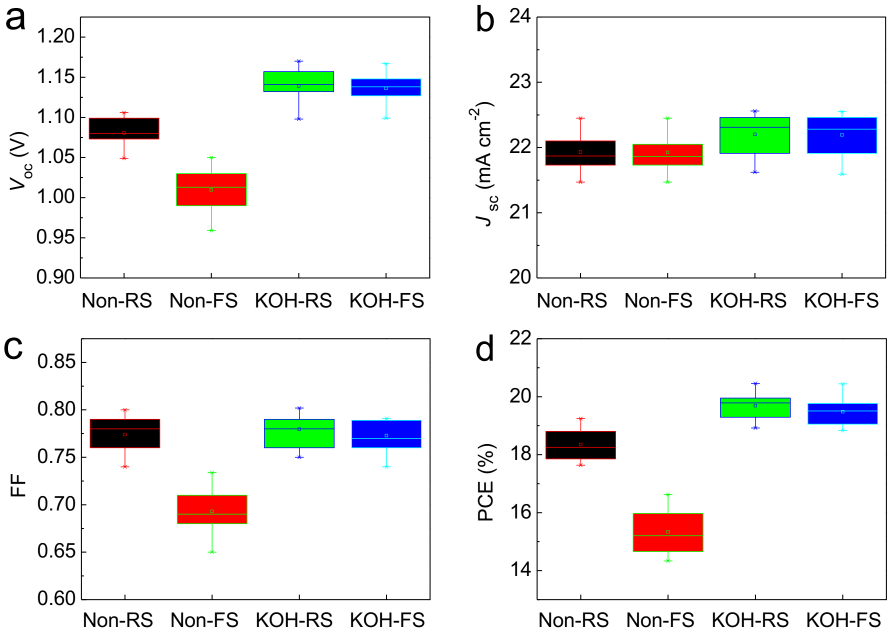

Supplementary Figure 8 Statistical distribution of the photovoltaic parameters for SnO_2 NCs based PSCs with or without 10 mM KOH treatment, respectively. Distribution of **a** V_{oc}, **b** J_{sc}, **c** FF, and **d** PCE

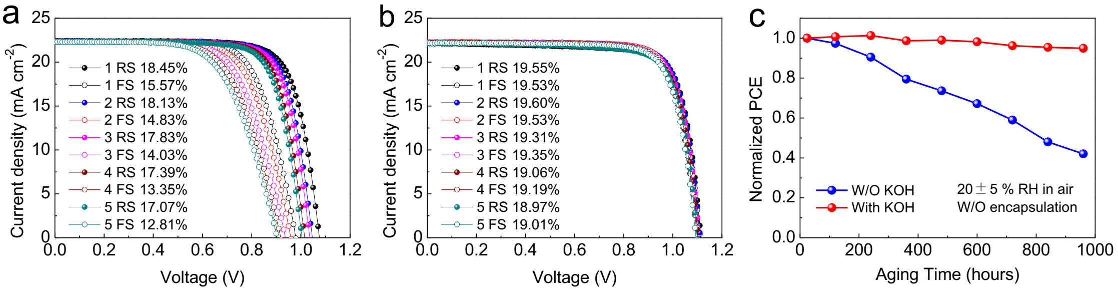

Supplementary Figure 9 a Continuous *J-V* scan of the SnO_2 NCs based PSCs, **b** Continuous *J-V* scan of the SnO_2 NCs based PSCs with 10 mM KOH treatment and **c** Stability test of the PSCs with/without KOH treatment for 30 days stored in ambient air without encapsulation (approximately 20 %RH)

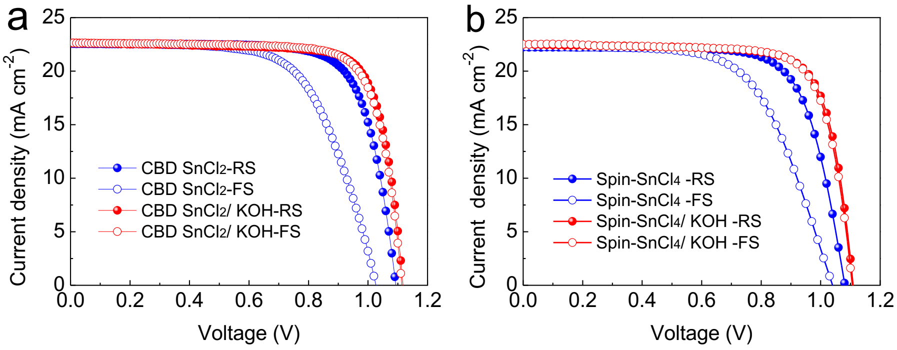

Supplementary Figure 10 Typical *J-V* curves of PSCs based on different SnO_2 films with or without 10 mM KOH treatment: **a** CBD $SnCl_2$ and **b** spin-coated $SnCl_4$

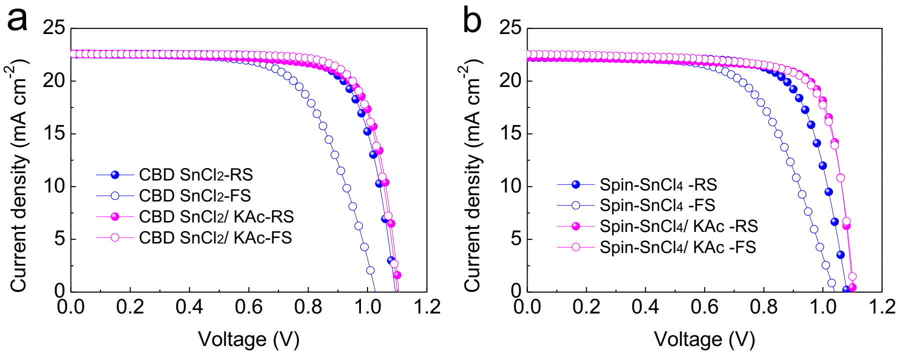

Supplementary Figure 11 Typical *J-V* curves of PSCs based on different SnO_2 films with or without 10 mM KAc treatment: **a** CBD $SnCl_2$ and **b** spin-coated $SnCl_4$

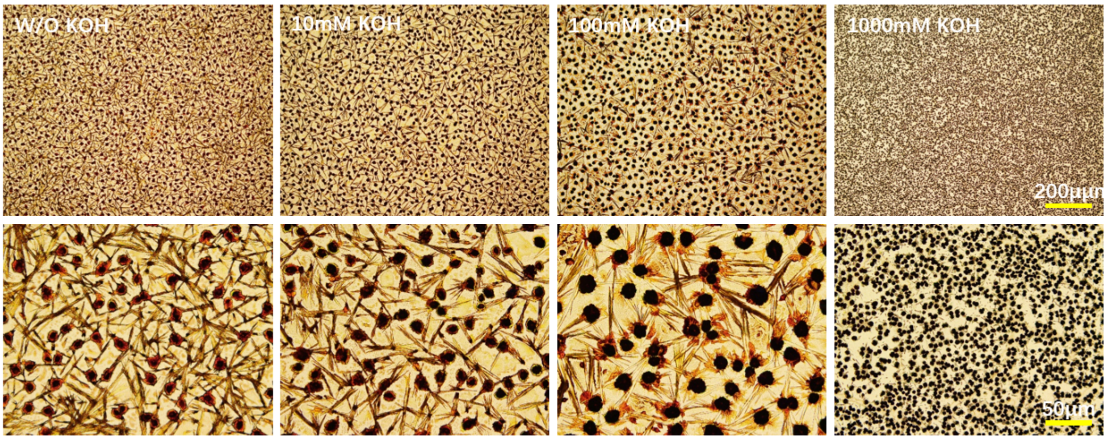

Supplementary Figure 12 Optical microscopies of the perovskite precursor deposited onto different concentration of KOH treated FTO substrates

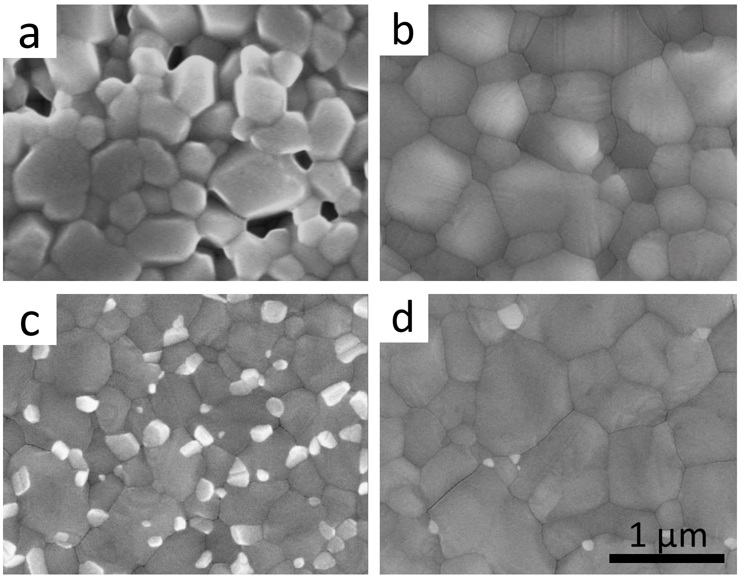

Supplementary Figure 13 SEM images of CsFAMA perovskite deposited on different SnO_2 NCs films. **a** Perovskite film deposited on pure SnO_2 NCs substrate without annealing, **b** perovskite film deposited on 10 mM KOH treated SnO_2 NCs substrate without annealing, **c** perovskite film deposited on pure SnO_2 NCs substrate with annealing, and **d** perovskite film deposited on 10 mM KOH treated SnO_2 NCs substrate with annealing

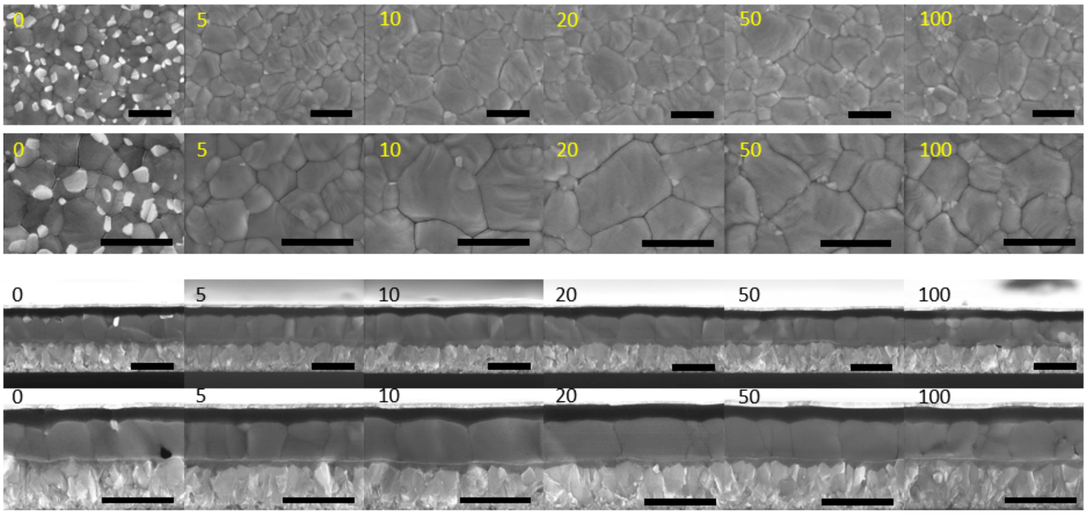

Supplementary Figure 14 SEM images and cross-section SEM images of CsFAMA perovskite deposited on SnO_2 NCs films with different concentration of KOH treatment. The scale bars are 1 μm

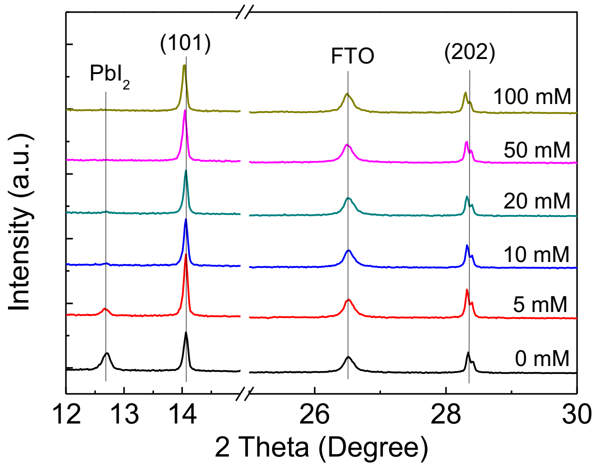

Supplementary Figure 15 XRD pattern of CsFAMA perovskite deposited on SnO_2 NCs films with different concentration of KOH treatment

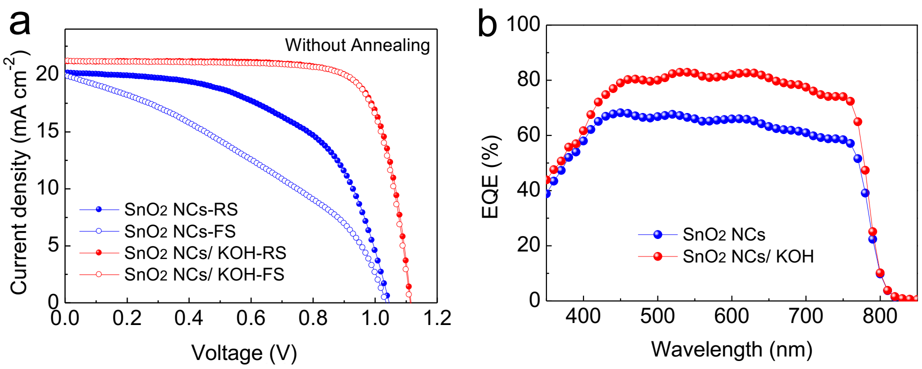

Supplementary Figure 16 Typical *J-V* curves of PSCs with annealing-free perovskites based on SnO_2 NCs films with or without 10 mM KOH treatment

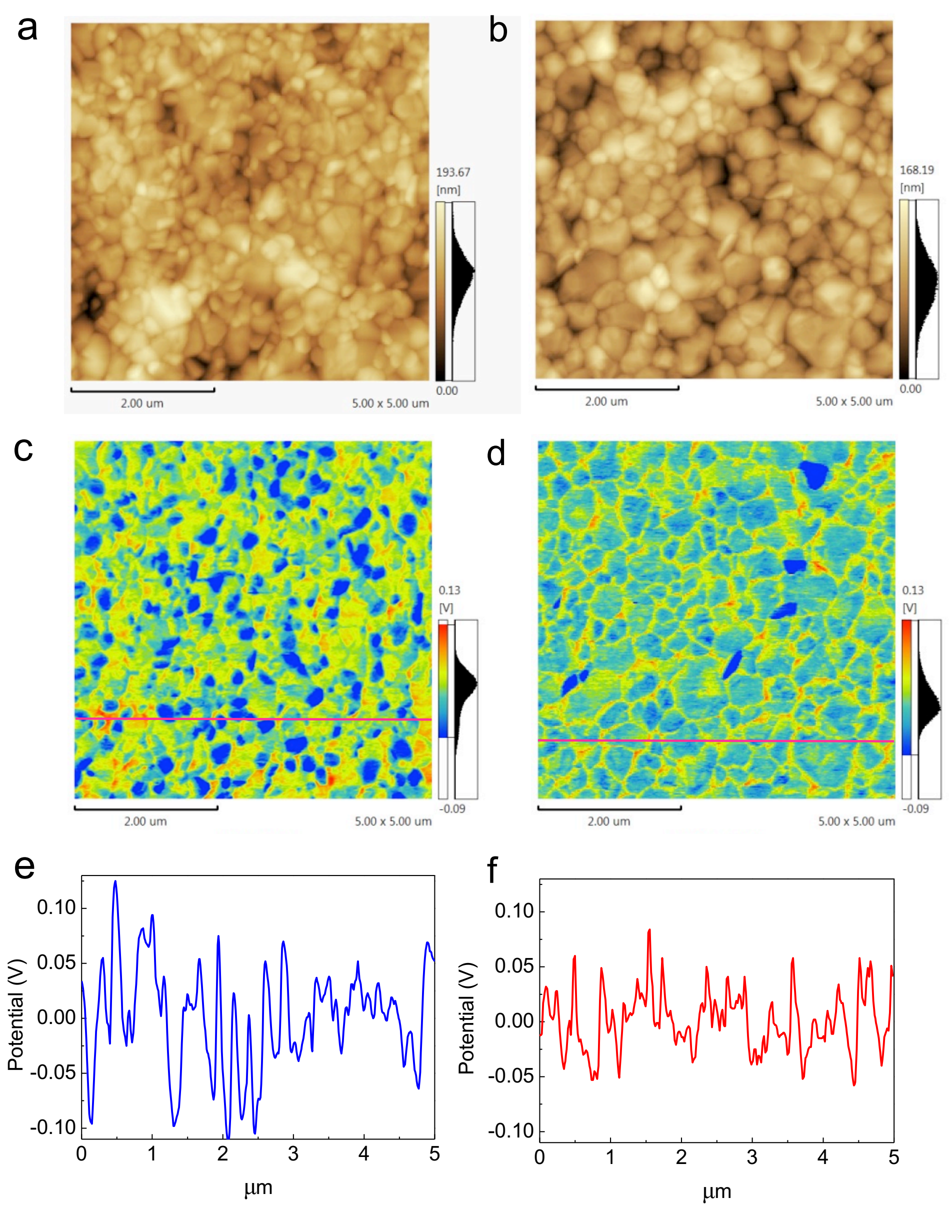

Supplementary Figure 17 AFM and KPFM images of CsFAMA perovskite deposited on SnO_2 NCs films with (**a**, **c**, **e**) or without (**b**, **d**, **f**)10 mM KOH treatment

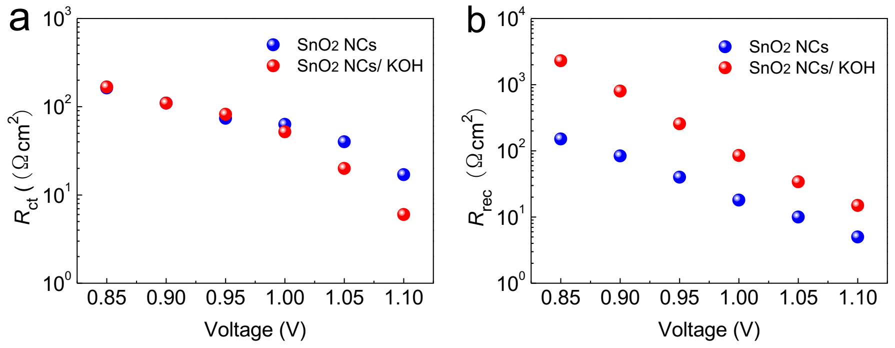

Supplementary Figure 18 a The contact resistance (R_{ct}) and **b** recombination resistance (R_{rec}) of devices at different biases

Supplementary Table 1 Literature review of hysteresis of the planar PSCs based on SnO_2 ETLs made by different methods

**Ref.**	**Preparation methods**	**Device structure**	**PCE (RS/FS)**	**HI (%)**	**Interface passivation**	**PCE (RS/FS)**	**HI (%)**
1	Spin-coating	ITO/Sb-doped $SnO_2/MAPbI_3$/Spiro-OMeTAD/Au	15.7%/13.2%	15.92	Sb-doped SnO_2	17.2%/16.2%	5.81
2	Spin-coating	FTO/Y-doped SnO_2/meso-$SnO_2/MAPbI_3$/ Spiro-OMeTAD/Au	13.38%/10.15%	24.14	Y-doped SnO_2	17.29%/16.97%	1.85
3	Spin-coating	FTO/Nb-doped $SnO_2/(FAPbI_3)_{0.85}(MAPbBr_3)_{0.15}$/Spiro-OMeTAD/Au	15.13%/11.12%	26.5	Nb-doped SnO_2	17.57%/12.75%	27.43
4	Spin-coating	FTO/Li:SnO_2/ $MAPbI_3$/Spiro-OMeTAD/Au	15.29%/13.55%	11.38	Li-doped SnO_2	18.2%/17.14%	5.82
5	CBD	FTO/Nb-doped $SnO_2/Cs_5(MA_{0.1}7FA_{0.83})_{95}Pb(I_{0.83}Br_{0.17})_3$/Spiro-OMeTAD/Au	19.70%	-2.08	Nb-doped SnO_2	20.50%	-1.39
6	Spin-coating	ITO/SnO_2:GQDs/$MAPbI_3$/Spiro-OMeTAD/Au	17.91%/15.84%	11.56	SnO_2:GQDs	20.31%/19.68%	3.1
7	PEALD	FTO/SnO_2/C60-SAM/$MA_{0.7}FA_{0.3}PbI_3$/Spiro-OMeTAD/Au	17.78%/16.33%	8.16	C60-SAM	19.28%/19.25	0.16
8	PEALD	FTO/SnO_2/C60-SAM/$MA_{0.7}FA_{0.3}PbI_3$/Spiro-OMeTAD/Au	16.26%/14.10%	13.28	C60-SAM	20.42%/20.29%	0.64
9	Spin-coating	FTO/SnO_2/PCBM/$MAPbI_3$/Spiro-OMeTAD/Au	16.53%/14.91%	9.8	PCBM	19.12%/18.10%	5.33
10	Spin-coating	FTO/SnO_2 QD/$MAPbI_3$/ Spiro-OMeTAD/Au	19.41%/17.62%	9.22	PCBM	20.26%/19.62%	3.16
11	Spin-coating	FTO/$SnO_2/(FAPbI_3)_{0.875}(CsPbBr_3)_{0.125}$/Spiro-OMeTAD/Au	16.66%/15.20%	8.76	K-doped Perovskite	19.40%/18.93%	2.42

12	CBD	$FTO/SnO_2/Kx(Cs_{0.05}FA_{0.80}MA_{0.15})_{(1-x)}Pb(I_{0.85}Br_{0.15})_3$/ Spiro-OMeTAD/Au	19.21%/15.52%	19.21	K-doped Perovskite	20.12%/20.56%	-2.19
13	Spin-coating	$ITO/SnO_2/(FAPbI_3)_{0.97}(MAPbBr_3)_{0.03}$/Spiro-OMeTAD/Au	—	—	Alfa-SnO_2	20.27%/20.54%	-1.33
14	Spin-coating	ITO/SnO_2/ $(FAPbI_3)_{1-x}(MAPbBr_3)_x$/Spiro-OMeTAD/Au	18.37%/19.41%	-5.66	Moderate Residual of PbI2	21.52%/21.20%	1.49
15	Spin-coating	$FTO/TiO_2/SnO_2/(FAPbI_3)_{0.85}(MAPbBr_3)_{0.15}$/PTAA/Au	11.13%	—	TiO_2/SnO_2 Bilayer	19.45%/18.10%	6.94
16	Spin-coating	$FTO/SnO_2/(FAPbI_3)_{0.85}(MAPbBr_3)_{0.15}$/Spiro-OMeTAD/Au	19.18%/17.10%	10.84	—	—	10.84
17	CBD	$FTO/SnO_2@TiO_2/(FAPbI_3)_{0.3}(MAPbI_3)_{0.7}$/Spiro-OMeTAD/Au	20.72%/17.15%	17.23	TiO_2/SnO_2 Bilayer	21.95%/20.59%	6.2
18	Spin-coating	$FTO/SnO_2/KCl/Cs_{0.05}FA_{0.81}MA_{0.14}PbI_{2.55}Br_{0.45}$/ Spiro-OMeTAD/Au,	17.3%/11.2%	35.26	KCl	20.5%/20.5	0
19	PLD	FTO/SnO_2/PCBM/$MAPbI_3$/Spiro-OMeTAD/Au	15.45%/13.18%	14.69	PCBM	17.03%/16.70%	1.94

Note: HI = $(PCE_{Reverse}-PCE_{Forward}) / PCE_{Reverse}$ [20]

Supplementary Table 2 The parameters derived from the *J-V* curves of rigid PSCs based on different Alfa-SnO_2 substrates

Devices	Sweep	V_{oc} (V)	J_{sc} (mA cm^{-2})	FF	PCE (%)
Alfa-SnO_2	RS	1.111	22.28	0.77	19.06
	FS	1.112	22.28	0.77	19.10
Water Alfa-SnO_2	RS	1.062	21.85	0.75	17.40
	FS	0.971	21.85	0.67	14.28
Water Alfa-SnO_2/KOH	RS	1.111	22.32	0.75	18.62
	FS	1.108	22.32	0.75	18.57

Supplementary Table 3 The parameters derived from the typical *J-V* curves of rigid PSCs based on different concentration of KOH treated SnO_2 substrates

Devices	Sweep	V_{oc} (V)	J_{sc} (mA cm^{-2})	FF	PCE (%)
SnO_2 NCs	RS	1.095	22.26	0.78	19.01
	FS	1.005	22.27	0.70	15.66
SnO_2 NCs/ KOH 5 mM	RS	1.124	22.37	0.77	19.29
	FS	1.113	22.37	0.76	18.82
SnO_2 NCs/ KOH 10 mM	RS	1.146	22.48	0.79	20.25
	FS	1.144	22.48	0.78	20.06
SnO_2 NCs/ KOH 20 mM	RS	1.146	22.40	0.78	20.07
	FS	1.146	22.41	0.77	19.91
SnO_2 NCs/ KOH 50 mM	RS	1.146	21.84	0.75	18.81
	FS	1.149	21.82	0.76	19.05
SnO_2 NCs/ KOH 100 mM	RS	1.145	21.58	0.74	18.16
	FS	1.150	21.55	0.75	18.58

Supplementary Table 4 The parameters derived from the *J-V* curves of rigid PSCs based on 10 mM KOH treated SnO_2 substrates without annealing

Devices	Sweep	V_{oc} (V)	J_{sc} (mA cm^{-2})	FF	PCE (%)
SnO_2 NCs	RS	1.045	20.16	0.56	11.74
	FS	1.035	19.94	0.37	7.63
SnO_2 NCs/KOH	RS	1.116	21.16	0.77	18.16
	FS	1.113	21.18	0.77	18.15

Supplementary Table 5 The fitted parameters of CsFAMA perovskite deposited on different SnO_2 substrates from TRPL spectra

Illuminate Direction	Perovskite side		Glass side	
Parameters	SnO_2 NCs	SnO_2 NCs/KOH	SnO_2 NCs	SnO_2 NCs/KOH
A_1	0.25	0.15	0.30	0.37
τ_1 (ns)	11.01	7.33	16.13	12.02
A_2	0.67	0.72	0.55	0.44
τ_2 (ns)	286.48	441.83	226.83	273.68
$A_1/(A_1+A_2)$	0.27	0.17	0.35	0.46

Note: $y = \mathrm{A1}\exp\left(-\frac{t}{\tau 1}\right) + \mathrm{A2}\exp\left(-\frac{t}{\tau 2}\right)$

Supplementary References

1 Bai, Y. *et al.* Low Temperature Solution-Processed Sb:SnO_2 Nanocrystals for Efficient Planar Perovskite Solar Cells. *ChemSusChem* **9**, 2686-2691 (2016).

2 Yang, G. *et al.* Reducing Hysteresis and Enhancing Performance of Perovskite Solar Cells Using Low-Temperature Processed Y-Doped SnO_2 Nanosheets as Electron Selective Layers. *Small* **13** (2017).

3 Ren, X. *et al.* Solution-Processed Nb:SnO_2 Electron Transport Layer for Efficient Planar Perovskite Solar Cells. *ACS Appl. Mater. Interfaces* **9**, 2421-2429 (2017).

4 Park, M. *et al.* Low-temperature solution-processed Li-doped SnO_2 as an effective electron transporting layer for high-performance flexible and wearable perovskite solar cells. *Nano Energy* **26**, 208-215 (2016).

5 Halvani Anaraki, E. *et al.* Low-Temperature Nb-Doped SnO_2 Electron-Selective Contact Yields over 20% Efficiency in Planar Perovskite Solar Cells. *ACS Energy Lett.* **3**, 773-778 (2018).

6 Xie, J. *et al.* Enhanced Electronic Properties of SnO_2 via Electron Transfer from Graphene Quantum Dots for Efficient Perovskite Solar Cells. *ACS Nano* **11**, 9176-9182 (2017).

7 Xiao, C. *et al.* Junction Quality of SnO_2-Based Perovskite Solar Cells Investigated by Nanometer-Scale Electrical Potential Profiling. *ACS Appl. Mater. Interfaces* **9**, 38373-38380 (2017).

8 Wang, C. *et al.* Understanding and Eliminating Hysteresis for Highly Efficient Planar Perovskite Solar Cells. *Adv.Energy Mater.* **7**, 1700414 (2017).

9 Ke, W. *et al.* Cooperative tin oxide fullerene electron selective layers for high-performance planar perovskite solar cells. *J. Mater. Chem. A* **4**, 14276-14283 (2016).

10 Yang, G. *et al.* Effective Carrier-Concentration Tuning of SnO_2 Quantum Dot Electron-Selective Layers for High-Performance Planar Perovskite Solar Cells. *Adv. mater.* **30**, e1706023 (2018).

11 Jung, K.-H., Seo, J.-Y., Lee, S., Shin, H. & Park, N.-G. Solution-processed SnO_2 thin film for a hysteresis-free planar perovskite solar cell with a power conversion efficiency of 19.2%. *J. Mater. Chem. A* **5**, 24790–24803 (2017).

12 Bu, T. *et al.* A novel quadruple-cation absorber for universal hysteresis elimination for high efficiency and stable perovskite solar cells. *Energy Environ. Sci.* **10**, 2509-2515 (2017).

13 Jiang, Q. *et al.* Enhanced electron extraction using SnO_2 for high-efficiency planar-structure HC(NH2)2PbI3-based perovskite solar cells. *Nat. Energy* **2**, 16177 (2016).

14 Jiang, Q. *et al.* Planar-Structure Perovskite Solar Cells with Efficiency beyond 21. *Adv. Mater.* **29** (2017).

15 Lee, Y. *et al.* Enhanced charge collection with passivation of the tin oxide layer in planar perovskite solar cells. *J.Mater. Chem. A* **5**, 12729-12734 (2017).

16 Dong, Q., Shi, Y., Zhang, C., Wu, Y. & Wang, L. Energetically favored formation of SnO_2 nanocrystals as electron transfer layer in perovskite solar cells with high efficiency exceeding 19%. *Nano Energy* **40**, 336-344 (2017).

17 Ding, B. *et al.* Low-temperature SnO_2-modified TiO_2 yields record efficiency for normal planar perovskite solar modules. *J. Mater. Chem. A* **6**, 10233-10242 (2018).

18 Liu, X. *et al.* Exploring Inorganic Binary Alkaline Halide to Passivate Defects in Low-Temperature-Processed Planar-Structure Hybrid Perovskite Solar Cells. *Adv. Energy Mater.*, 1800138 (2018).

19 Chen, Z. L. *et al.* Bulk heterojunction perovskite solar cells based on room temperature deposited hole-blocking layer: Suppressed hysteresis and flexible photovoltaic application. *J. Power Sources* **351**, 123-129 (2017).

20 Wang, S. *et al.* Sequential Processing: Spontaneous Improvements in Film Quality and Interfacial Engineering for Efficient Perovskite Solar Cells. *Solar RRL* **2**, 1800027 (2018).
